# Sustained and Transient Vestibular Systems: A Physiological Basis for Interpreting Vestibular Function

**DOI:** 10.3389/fneur.2017.00117

**Published:** 2017-03-30

**Authors:** Ian S. Curthoys, Hamish G. MacDougall, Pierre-Paul Vidal, Catherine de Waele

**Affiliations:** ^1^Vestibular Research Laboratory, School of Psychology, The University of Sydney, Sydney, NSW, Australia; ^2^Cognition and Action Group, CNRS UMR8257, Centre Universitaire des Saints-Pères, University Paris Descartes, Paris, France; ^3^ENT Department, Salpêtrière Hospital, Paris, France

**Keywords:** vestibular, utricular, sound, vibration, otolith, vestibular-evoked myogenic potential, vestibulo-ocular reflex, vestibular-evoked myogenic potentials

## Abstract

Otolithic afferents with regular resting discharge respond to gravity or low-frequency linear accelerations, and we term these the static or sustained otolithic system. However, in the otolithic sense organs, there is anatomical differentiation across the maculae and corresponding physiological differentiation. A specialized band of receptors called the striola consists of mainly type I receptors whose hair bundles are weakly tethered to the overlying otolithic membrane. The afferent neurons, which form calyx synapses on type I striolar receptors, have irregular resting discharge and have low thresholds to high frequency (e.g., 500 Hz) bone-conducted vibration and air-conducted sound. High-frequency sound and vibration likely causes fluid displacement which deflects the weakly tethered hair bundles of the very fast type I receptors. Irregular vestibular afferents show phase locking, similar to cochlear afferents, up to stimulus frequencies of kilohertz. We term these irregular afferents the transient system signaling dynamic otolithic stimulation. A 500-Hz vibration preferentially activates the otolith irregular afferents, since regular afferents are not activated at intensities used in clinical testing, whereas irregular afferents have low thresholds. We show how this sustained and transient distinction applies at the vestibular nuclei. The two systems have differential responses to vibration and sound, to ototoxic antibiotics, to galvanic stimulation, and to natural linear acceleration, and such differential sensitivity allows probing of the two systems. A 500-Hz vibration that selectively activates irregular otolithic afferents results in stimulus-locked eye movements in animals and humans. The preparatory myogenic potentials for these eye movements are measured in the new clinical test of otolith function—ocular vestibular-evoked myogenic potentials. We suggest 500-Hz vibration may identify the contribution of the transient system to vestibular controlled responses, such as vestibulo-ocular, vestibulo-spinal, and vestibulo-sympathetic responses. The prospect of particular treatments targeting one or the other of the transient or sustained systems is now being realized in the clinic by the use of intratympanic gentamicin which preferentially attacks type I receptors. We suggest that it is valuable to view vestibular responses by this sustained-transient distinction.

## Key Concepts

Vestibular afferents with regular resting discharge constitute a system for signaling sustained vestibular stimuli, such as maintained head tilts.

Vestibular afferents with irregular resting activity constitute a system for signaling transient vestibular stimuli.

Otolith irregular afferents originate from a specialized region of the otolithic maculae called the striola and form calyx synapses on type I receptors which have fast membrane dynamics.

Otolith irregular afferents are selectively activated by high-frequency (~500 Hz) low-intensity BCV and ACS. Regular otolithic afferents do not respond to these stimuli at comparable levels.

Ototoxic antibiotics selectively affect type I receptors and thus the transient system.

The 500-Hz vibration causes eye movements in humans, and the preparatory myogenic potentials for these movements are the ocular VEMPs which index the activity of the transient system.

Vestibular nucleus neurons exhibit transient or sustained responses, as in the periphery.

## Introduction

The early recordings of the response of cat single vestibular nucleus neurons to angular acceleration established that different neurons had very different response patterns to identical angular acceleration stimuli (1–3). Some neurons showed a sustained response to maintained stimuli (termed tonic neurons), whereas others showed a transient response to the same stimulus (termed kinetic neurons). These results were confirmed and extended in later research which also showed a similar distinction applied to primary semicircular canal (4–6) and otolithic neurons (7–9). That research and later work showed how these characteristics were associated with the regularity of resting discharge of primary afferent neurons—neurons with regular resting discharge showing sustained (tonic) responses to maintained stimuli, whereas neurons with irregular resting discharge showed transient (kinetic or phasic) responses to the same stimuli (10). There is a continuum of regularity, and we will use the terms sustained and transient for convenience to refer to the ends of this continuum.

This review shows how recent evidence about otolithic responses to sound and vibration demonstrates the value of applying this distinction of sustained and transient from the receptors to the behavioral responses. We suggest it is a valuable principle for interpreting the results of vestibular functional tests—focusing attention on different aspects of the response rather than just treating the whole response as uniform. This is especially clear with the otoliths, but even with the canals it is useful to distinguish between responses to the onset of an acceleration, as opposed to responses during maintained accelerations. The paper will not cover particular areas in great detail since it has already been done in many other reviews to which the reader is referred. Instead, we aim to bring together evidence from physiology to highlight the relevance of this evidence for understanding vestibular function testing as used clinically. The first part of this review covers physiological evidence and the second part covers the application of the sustained-transient principle to vestibular responses.

## Physiological Data

### Peripheral Vestibular Physiology

The regularity of resting discharge of vestibular primary afferents is associated with a range of characteristics, such as conduction velocity, axon thickness, and a range of response dimensions, such as gain to acceleration, sensitivity to electrical vestibular stimulation [including so-called galvanic vestibular stimulation (GVS)—DC or low-frequency electrical stimulation of the sense organs]. While afferents from all vestibular sense organs are activated by GVS at approximately equal thresholds (11) irregular afferents from each sense organ have a significantly lower threshold for GVS activation than regular neurons.

Angular and linear acceleration of the whole animal have been the usual stimuli in studies of vestibular physiology, and it has been shown in a number of species that regular and irregular neurons have different frequency responses—with irregular afferents (both canal and otolithic) typically having an increased gain and increasing phase lead with increasing frequency—interpreted as showing that irregular afferents are responsive to both acceleration and change in acceleration (jerk). The characteristics of regular and irregular neurons have been summarized by Goldberg’s definitive review of vestibular afferent diversity (10).

**Figure 1:**
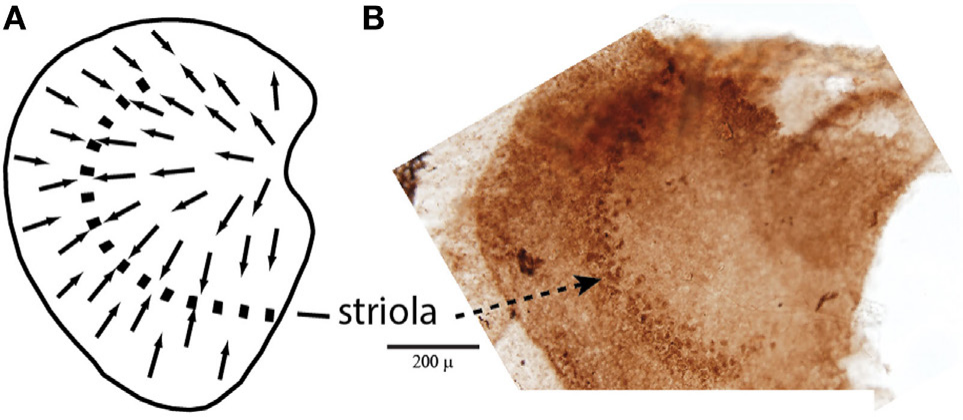
**(A)** Schematic representation of a dorsal view of the whole guinea pig utricular macula, with the arrows showing the polarization of receptor hair cells. The dashed line is the line of polarity reversal which used to be called the striola, but more recently it is recognized that the striola is a band of receptors as shown in the adjacent whole mount. **(B)** To show a corresponding whole mount of a guinea pig utricular macula treated by calretinin—the band of cells comprising the striola is clearly visible (54). Reprinted from Ref. (21), Copyright (2012), with permission from Elsevier.

In summary, first-order vestibular neurons with regular resting discharge comprised bouton and dimorphic neurons which synapse on the barrel-shaped type II receptors mainly in the peripheral zone of the cristae and in the extrastriolar zone of the otolithic maculae. They are characterized by thin or medium-sized, slow conducting axons and with a low sensitivity to head rotation and relatively low sensitivity to GVS. Irregular first-order vestibular neurons comprised calyx and dimorphic neurons, which innervate the central cristae and the striolar zones of the otolithic maculae, synapsing predominantly on the amphora-shaped type I receptors (Figure 1). They are characterized by large- or medium-sized fast conducting axons. Their sensitivity to GVS is on average six times higher than that of the regular afferents (11, 12).

**Figure 2:**
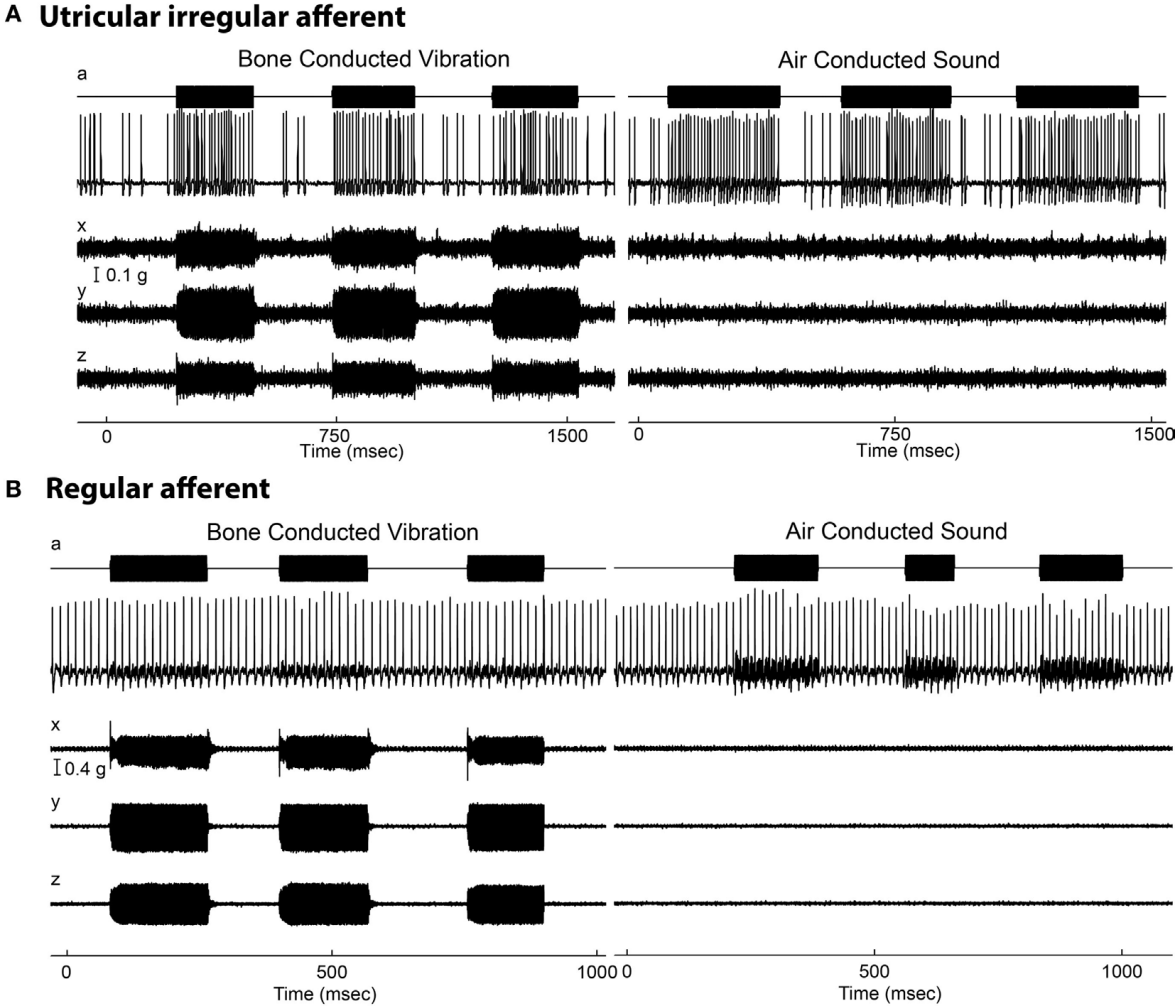
**Resting discharge pattern and response to stimulation of an irregular and a regular afferent**. **(A)** Time series of an irregular otolith neuron during stimulation by 500 Hz bone-conducted vibration (BCV) and air-conducted sound (ACS). The top trace (a) shows the command voltage, indicating when the stimulus is on. The second trace shows the action potentials by extracellular recording. The three bottom traces (*x, y, z*) show the triaxial accelerometer recording of the stimulus. The left panel is an example of response to BCV stimulation and the right of the response to ACS stimulation of the same neuron, showing it is clearly activated by both stimulus types. Note the scale of stimulus intensity in *g* at the left margin between traces *x* and *y*. The irregular resting discharge is seen before stimulus onset, followed by a large increase in firing during both BCV and ACS. **(B)** Time series of a regular semicircular canal neuron during stimulation by BCV and ACS as above. The regular discharge is seen before the stimulus onset. The stimuli are far stronger than in panel **(A)**, but there is no evidence of activation of this regular neuron by these strong stimuli. From Ref. (19), Curthoys and Vulovic, © Springer-Verlag, 2010, reproduced with permission of Springer.

In addition, some vestibular afferents show a sensitive response to sound and vibration (13–15). Recent research has extended this account (16–21). The major result is that the otolith irregular neurons originating from a special band of receptors on the otolithic macula called the striola (Figure 2) respond to both air-conducted sound (ACS) and bone-conducted vibration (BCV) up to very high frequencies (>1,000 Hz), while regular neurons—mainly from extrastriolar areas—show modest or absent responses to such stimuli (see Figure 2).

**Figure 3:**
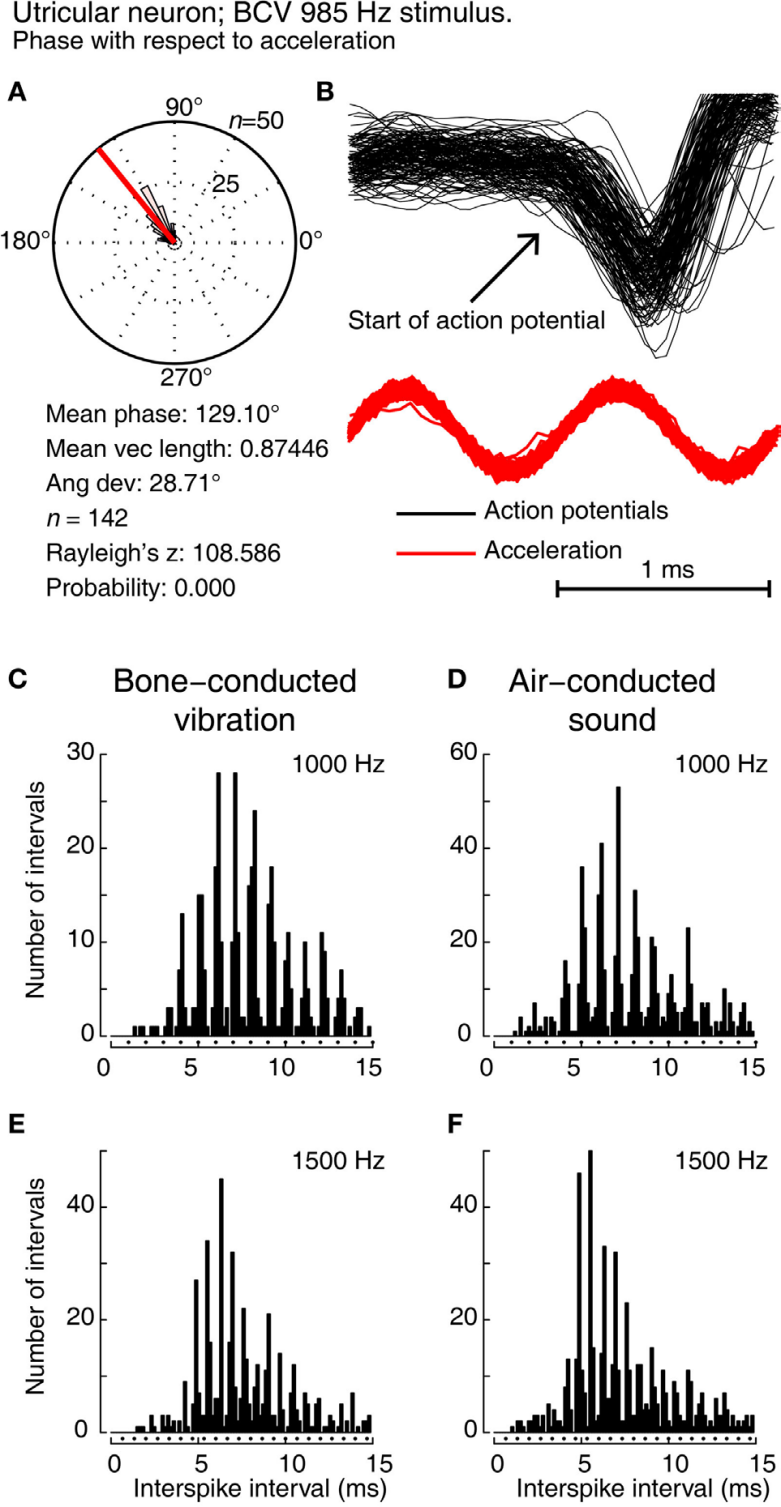
**Showing phase locking of a single utricular neuron**. **(A,B)** Time series of successive action potentials of the neuron to bone-conducted vibration (BCV) at 985 Hz. Panel **(B)** shows 142 action potentials superimposed and the onset of the action potential is shown by the arrow. The red trace shows the *x* channel of the 3D linear accelerometer. Panel **(A)** shows the circular histogram of the phases of the action potentials clustered around a mean of 129.1°, with angular deviation 28.7°. The test of circular uniformity, Rayleigh’s *z*, is highly significant showing the probability of a uniform phase distribution is <0.001. The neuron misses some cycles, but when it fires is locked to the stimulus waveform. **(C–F)** Histograms of interspike intervals to show phase locking in the same utricular afferent neuron in guinea pig at two high frequencies of BCV **(C,E)** and air-conducted sound (ACS) stimuli **(D,F)**. The bin width is 0.16 ms. The dots below each histogram show integral multiples of the period for the given stimulus frequency. The clustering around these integral multiples demonstrates phase locking at both frequencies. This figure is a replotting of the histograms of the response of neuron 151011, which has been published as Figure 10 of Curthoys et al. (20). Reprinted from Ref. (20), © 2016, with permission of Elsevier.

Detailed analysis of the timing of the action potentials of irregular afferents evoked by sound or vibration show they are phase locked to individual cycles of the ACS (up to 3,000 Hz) or BCV stimulus (up to 2,000 Hz) (Figure 3) (20). Phase locking means that the exact timing of the action potential is locked to a particular phase angle (or a narrow band of phase angles) of the stimulus waveform at that frequency (Figure 3). It does not mean the cell generates an action potential once per cycle up to thousands of Hertz, but that the moment when the cell fires is locked to the particular narrow band of phase angles of the stimulus waveform at that frequency. A vestibular afferent may miss many cycles, but when it does fire it is locked to the phase angle of the stimulus waveform. The phase locking of vestibular afferents is similar to the well-documented phase locking of cochlear afferents to ACS, known since the study of Rose et al. (22).

In stark contrast, regular neurons (canal or otolith) are not activated by ACS or BCV stimuli—at least in response to stimulus levels used in clinical studies of human otolithic function (see Figure 2B)—and so do not show phase locking. As the example in Figure 2 shows, regular neurons simply continue to fire at their resting rate during stimuli which are much more intense than those which cause activation of irregular neurons. Irregular afferents have very low thresholds to BCV—at or below the levels needed for auditory brainstem response threshold. McCue and Guinan (13, 23, 24) showed that irregular saccular afferents were activated by ACS, and Murofushi et al. (14, 25, 26) reported that saccular afferents could be activated by high intensity ACS click stimuli. These results have been confirmed years later (17, 20). Curthoys et al. (18) tested the specific, sensitive response of irregular utricular neurons to 500 Hz BCV in guinea pig and found that very few utricular regular afferents were activated by high intensity ACS or BCV stimulation. That has also been confirmed in rat: where few regular afferents are activated even by intense ACS, although ACS is an effective stimulus for irregular neurons (27, 28). It has been shown that both saccular and utricular irregular afferents are activated by both 500 Hz ACS and BCV (21).

This clear phase locking of irregular vestibular afferents to such high frequencies of ACS and BCV stimulation has posed questions about how vestibular hair cell transduction mechanisms operate at such high frequencies. Vestibular responses are usually thought of as being for stimuli to a few tens of Hertz, not to stimuli of over 1,000 Hz. Furthermore, the fact that irregular and regular afferents have such very different responses to sound and vibration raises the possibility of the use of sound and vibration to probe the relative contribution of irregular (transient) and regular (sustained) otolithic afferents to various physiological and even behavioral responses, and thus the likely functional roles of these classes of afferents. One other question is of the mechanisms responsible for the regularity of resting discharge, which is now clearly established as being due to membrane characteristics of the afferent neurons (29–31).

In these studies, the usual result is that in animals with intact bony labyrinths, afferent neurons (either regular or irregular) from semicircular canals are not activated or only weakly activated by very intense stimulus levels of 500 Hz BCV and ACS (18, 27, 28). However, there are drastic changes in neural response after a small hole is made in the bony wall of the semicircular canal (even just 0.1 mm diameter in the case of the guinea pig). This opening, called a dehiscence or a semicircular canal dehiscence (SCD) results in a previously unresponsive irregular canal afferent being activated by sound and vibration at low stimulus levels (32, 33). The evidence is that the SCD decreases the impedance of the labyrinth and so, it is argued, acts to increase the fluid displacement sufficiently to deflect the receptors.

More recently, it has been found that even in animals with intact bony labyrinths, irregular canal afferents can be activated by very low-frequency BCV and indeed phase lock to 100 Hz BCV (17). However, the response of these irregular canal afferents declines with increasing BCV frequency, and so these irregular canal afferents are not activated at 500 Hz BCV even at high stimulus levels (17, 34, 35). In light of this evidence, we conclude 500 Hz BCV is a selective stimulus for irregular otolithic neurons. It is probable that this activation of canal afferents by such low-frequency BCV is the neural mechanism responsible for the clinical test of vestibular function—skull vibration-induced nystagmus (35, 36).

#### Mechanism

How can vestibular receptors and irregular otolithic afferents respond to such very high frequencies? Textbook schematic diagrams of the cristae and otolithic maculae give the impression that each vestibular sense organ is a uniform structure with receptor hair cells of similar height. It is now clear that is the very opposite of what is the case. Each macula and each crista shows complex anatomical differentiation across the surface. The receptor types are differentially distributed with predominantly type I receptors at the striola (37). The extrastriolar receptors appear to be more tightly tethered to the otoconial membrane (38) than are the striolar receptors. Receptors across the maculae (and cristae) are not of uniform height: at the striola of the maculae and at the crest of the cristae, the receptor hair bundles are shorter (and stiffer) than in the extrastriolar areas. Remarkably, this height difference can even be seen in Hunter-Duvar’s scanning electron micrograph of the whole utricular macula of the chinchilla (39). Morphological evidence shows apparently looser tethering of the hair bundles of striolar receptors to the overlying otolithic membrane in comparison with comparable receptors in the periphery of the macula (extrastriolar receptors) (38–44).

Most models of otolithic stimulation model displacement of the gelatinous otoconial membrane with otoconia adherent to its upper surface in response to linear acceleration stimulation, e.g., Ref. (45, 46). In such models, frequencies in the kilohertz range are beyond the upper mechanical cutoff of the system. Nevertheless, it is clear from the neural recordings that action potentials in individual neurons do respond and are phase locked to a narrow band of phases for stimulus frequencies even as high as 3,000 Hz (Figure 3). Phase locking of irregular vestibular afferents shows that every single cycle of the stimulus waveform is the adequate stimulus for the receptor–afferent complex. For this to happen at kilohertz frequencies, the receptor membrane and calyx membrane must have extremely fast dynamics, and indeed, the very fast dynamics of vestibular type I receptors and calyx membranes have been shown beautifully by the studies of Songer and Eatock (47). They used intracellular recording of receptor and calyx potentials in response to mechanical displacement of the hair bundle at very high frequencies and demonstrated the very fast membrane and synaptic dynamics of type I vestibular receptors and calyx afferents.

In light of this phase locking up to such high frequencies, we have suggested that the hair bundles of the striolar otolithic receptors are deflected by each cycle of fluid displacement caused by the stapes pumping into the fluid-filled inner ear (33). This fluid displacement is small but vestibular receptors have great sensitivity: the maximum sensitivity of the hair bundles of vestibular receptors is around 0.40° of cilia deflection, similar to the value for cochlear receptors (0.39°) (48), caused by just a few nanometers of fluid displacement.

We (33) have put forward the following account. Striola receptors are predominantly amphora-shaped type I receptors (37) and have short stiff hair bundles (44, 49), apparently loosely attached to the overlying otolithic membrane (38). This fluid motion within the fluid-filled hole in the gel-filament layer of the otolithic membrane produces a drag force on the hair bundle, causing it to deflect. The fluid environment is so viscously dominated (Reynold’s Numbers of 10^−3^ to 10^−2^) that bundles move instantaneously with any fluid movement. In other words, this coupling of fluid motion to hair bundle is so strong that the hair bundle displacement follows the fluid displacement almost exactly (17). Thus, fluid displacement is synonymous with hair bundle displacement.

**Figure 4:**
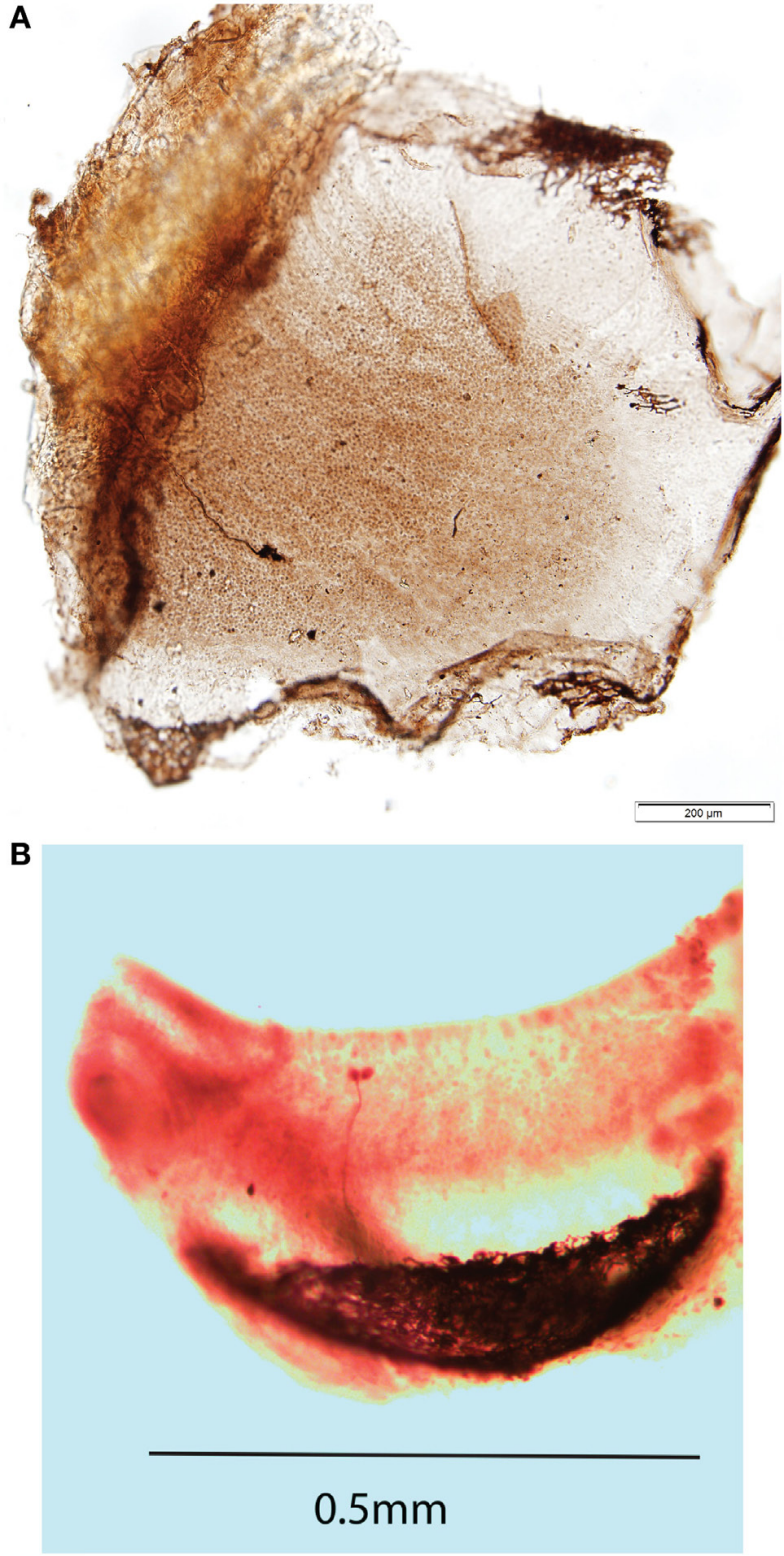
**(A)** Direct dorsal view of a whole mount of a guinea pig utricular macula showing one neuron labeled by neurobiotin activated by both 500 Hz bone-conducted vibration (BCV) and air-conducted sound (ACS). The labeled axon terminates on three calyx endings which envelop the whole type I receptor(s), but in addition there are small bouton endings, so this axon is strictly a dimorphic afferent. The calyx receptors are in or close to the striolar area of the utricular macula. The scale bar is 200 μm. Reprinted from Ref. (21), Copyright (2012), with permission from Elsevier. **(B)** Whole mount of the crista of the anterior canal of a guinea pig showing an afferent fiber labeled by neurobiotin which forms two calyx endings enveloping two type I receptors at the crest of the crista. This neuron was unresponsive to ACS prior to a semicircular canal dehiscence, but showed activation and phase locking to 985 Hz ACS after dehiscence.

Complementing that anatomical evidence is physiological evidence from recordings of primary otolithic afferent neurons originating from striola type I receptors as shown by neurobiotin labeling (21) (Figure 4A), these afferents have irregular resting discharge and are activated by ACS and BCV up to very high frequencies. There is evidence that it is the striolar receptor hair cells (probably mainly type I receptors) which respond to frequencies far higher than modeling of canal or otolith mechanics indicates. This account is indirectly confirmed by the effect of SCD on the response of irregular canal afferents from the crest of the crista (17). Prior to the SCD, these neurons do not respond to ACS or BCV at the levels used in human clinical testing, whereas after the SCD [which acts to increase fluid displacement (50, 51)] these same irregular afferents show strong phase-locked activation to the same stimulus (33) (Figure 4B).

Why is it that regular afferents are not responsive to vibration and sound at such high frequencies? One possible reason is that regular neurons synapse on few type I receptors mainly in extrastriolar areas, whereas their largest number of synapses are usually on multiple extrastriolar type II receptors with long cilia apparently more tightly tethered to the overlying gel-filament layer (38). In this way, fluid displacement would be less likely to activate type II receptors because, instead of projecting into holes in the overlying membrane, the hair bundles project into the gel-filament layer or cupula, which limits the deflection of the receptors.

#### Mechanism—Summary

There are two issues: (1) the regularity of resting discharge and (2) the response to sound and vibration determined by receptor mechanisms. These are two different aspects of the afferent response, and the evidence is that they are determined by different factors.

(1) Recent evidence shows that regularity of vestibular afferent resting discharge is due to membrane characteristics (29–31). That has been most convincingly shown by recent experiments blocking specific membrane channels. This is the empirical evidence—the regularity of afferent discharge is due to channels of the afferent membrane and not the receptors. Neurobiotin labeling has shown that the irregular afferents which respond to these stimuli form calyx synapses on striolar type 1 receptor cells, not on type I extrastriolar receptors (20, 21). However, the receptor type does not determine the regularity of resting discharge: fish only have type II receptors but they have both regular and irregular afferents (52).
(2) We (33) have put forward the hypothesis that the short stiff hair bundles of these striolar receptors are deflected by fluid displacement once per stimulus cycle so the calyx-bearing afferents of striolar irregular afferents are activated once per cycle, resulting in phase-locked action potentials up to such high frequencies. Hair cell height and stiffness have been demonstrated to vary across the macula surface (39, 49, 53–55). The cells which do show high-frequency phase locking are from calyx-bearing afferents originating from the striola (21) with shorter stiffer receptor hair cells. Afferents from extrastriolar areas do not show high-frequency phase locking. With only type II receptors, fish also show phase locking, but to much lower frequencies (~400 Hz), and it may be that the type I receptor calyx combination allows the very high-frequency phase locking found in guinea pigs.

### Central

The sustained–transient distinction is clear at the level of the primary afferent, whereas these complementary afferent systems would appear to be lost centrally (10, 56–58). In those studies, it was found that regular and irregular afferents projected to second-order vestibular neurons and showed overlapping projections—such that many second-order neurons received input from both regular and irregular afferents. Different vestibular nucleus neurons showed different strengths of irregular and regular projections. It appeared that the sustained and transient systems from the periphery were not established centrally (57, 58).

**Figure 5:**
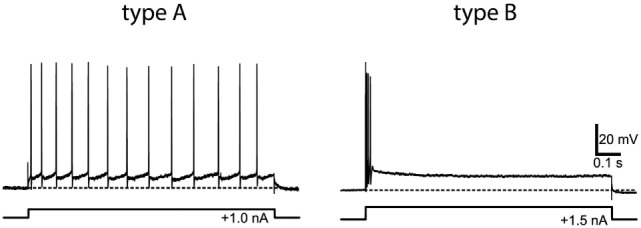
**Example of the response of type A (tonic) and type B (phasic) frog vestibular nucleus from *in vitro* whole frog brain preparation to the same step of current**. Reprinted from Straka et al. (64), with permission.

However, *in vitro* physiological studies, recording from vestibular nucleus neurons in slice preparations, have shown that at the vestibular nucleus there is clear evidence of different neurons with very different temporal responses to identical stimuli (injected current in these cases, rather than angular or linear acceleration), and that these neurons can be characterized by the same sustained–transient distinction as for primary afferents. Type A neurons show tonic characteristics—maintained firing to a step of injected current—whereas type B neurons show a brief high-frequency burst of spikes at stimulus onset (see Figure 5). These patterns have been established in frog and in mammals and echo the phasic-tonic distinction of Shimazu and Precht (2) in cat vestibular nucleus neurons. In frog, there are two well-defined populations of neurons with very different membrane properties and response patterns, and these correspond well to the transient and sustained classes. One approach has been to consider these matters from the point of view of filter characteristics, which is just another way of schematizing sustained (low-pass filter) and transient (high-pass filter) systems.

These results from *in vitro* studies (59) and from frogs (60–66) have been shown to apply also in mammalian vestibular system. Type A and B neurons previously found in brainstem slices were identified in the guinea pig whole-brain preparation, and these neurons receive direct inputs from the vestibular nerve. Type A and B vestibular nucleus neurons differ in their intrinsic membrane properties which are responsible for the very different response patterns shown in Figure 5 (62, 67) and for the different filtering properties (61, 68).

## Sustained and Transient Aspects of Vestibular Responses

### Tools for Probing the Functional State of the Sustained and Transient Systems

The evidence from physiology reviewed above establishes the sustained and transient categories of vestibular neural responses and provides tools for exploring the sustained and transient aspects of vestibular responses and how pathology may affect them. Using these tools, it is possible to identify sustained and transient aspects of behavioral responses. However, is this quest valuable for evaluating vestibular responses? We consider it is, because whereas some disorders affect all afferents it is likely that different treatments (e.g., gentamicin) and disorders will differentially affect the sustained and transient systems.

#### Acceleration Frequency

The frequency of the acceleration stimulus is one such tool. Sustained and transient systems have different frequency responses to acceleration stimulation (10)—the irregular neurons of both canals and otoliths showing a high-frequency preference, which has been interpreted as jerk sensitivity.

#### Galvanic

Galvanic stimulation *via* surface electrodes on the mastoids has been used in a very large number of studies of human vestibular responses. GVS is a complex stimulus which acts on all vestibular sense organs (11). It had been argued that GVS acts only on the afferent axon at the “spike trigger zone,” but the careful experiments of Gensberger et al. (69) show that GVS acts on both receptors and the axon. While irregular afferents do have a lower threshold for galvanic stimulation compared to regular neurons (11, 12), the numerical difference is not large and the variability between neurons is considerable. In principle, low-current, short-duration galvanic stimulation should preferentially activate irregular axons, and indeed, this stimulus is effective for evoking a myogenic response which, in light of the above evidence, we consider is a transient vestibular response—vestibular-evoked myogenic potentials (VEMPs)—discussed below (70, 71).

#### Vibration

The very different otolithic afferent responses to vibration mean that vibration is a particularly powerful tool in differentiating sustained and transient responses. Otolithic irregular afferent neurons are activated by high frequencies of vibration, whereas otolithic regular neurons are not activated at intensities used in usual clinical practice. Some caution is needed since recent results have shown that low-frequency BCV is not selective for otoliths: even in animals with intact bony labyrinths, 100-Hz BCV activates canal afferents (17). But the response of these canal neurons to BCV declines as the BCV frequency is increased, so that at 500 Hz there is no detectable activation at intensities which are used clinically. In light of this, we consider it reasonable to conclude that if high-frequency vibration (e.g., 500 Hz) elicits a response, then it is due to the action of the irregular otolithic afferents—the otolithic transient system, originating predominantly from type I receptors at the striola.

#### Vestibular-Evoked Potentials (VsEP)

Brief pulses of linear acceleration cause a short latency-evoked potential (called a VsEP) recorded in a variety of species with a variety of recording montages (72–76). Control experiments after cochlea ablation have shown that the VsEP is not due to the cochlea, but is a neural response to otolithic activation (77). The VsEP would appear to be due to the otolithic transient system, since it is change in linear acceleration (jerk) which is the adequate stimulus for the VsEP (78).

#### Gentamicin

Animal studies have shown that the type I receptors are more vulnerable to the effects of ototoxic antibiotics than are the type II receptors (79–81). In human patients, gentamicin is administered for therapeutic reasons either systemically or more commonly by intratympanic injection [intratympanic gentamicin (ITG)]. On the above account, it follows that gentamicin should affect the transient system and leave the sustained system relatively unaffected. As we show below, the responses to ITG are now identifying the differences between the sustained and transient systems.

## Sustained and Transient Aspects of Behavioral Responses

### Otolithic Responses

**Figure 6:**
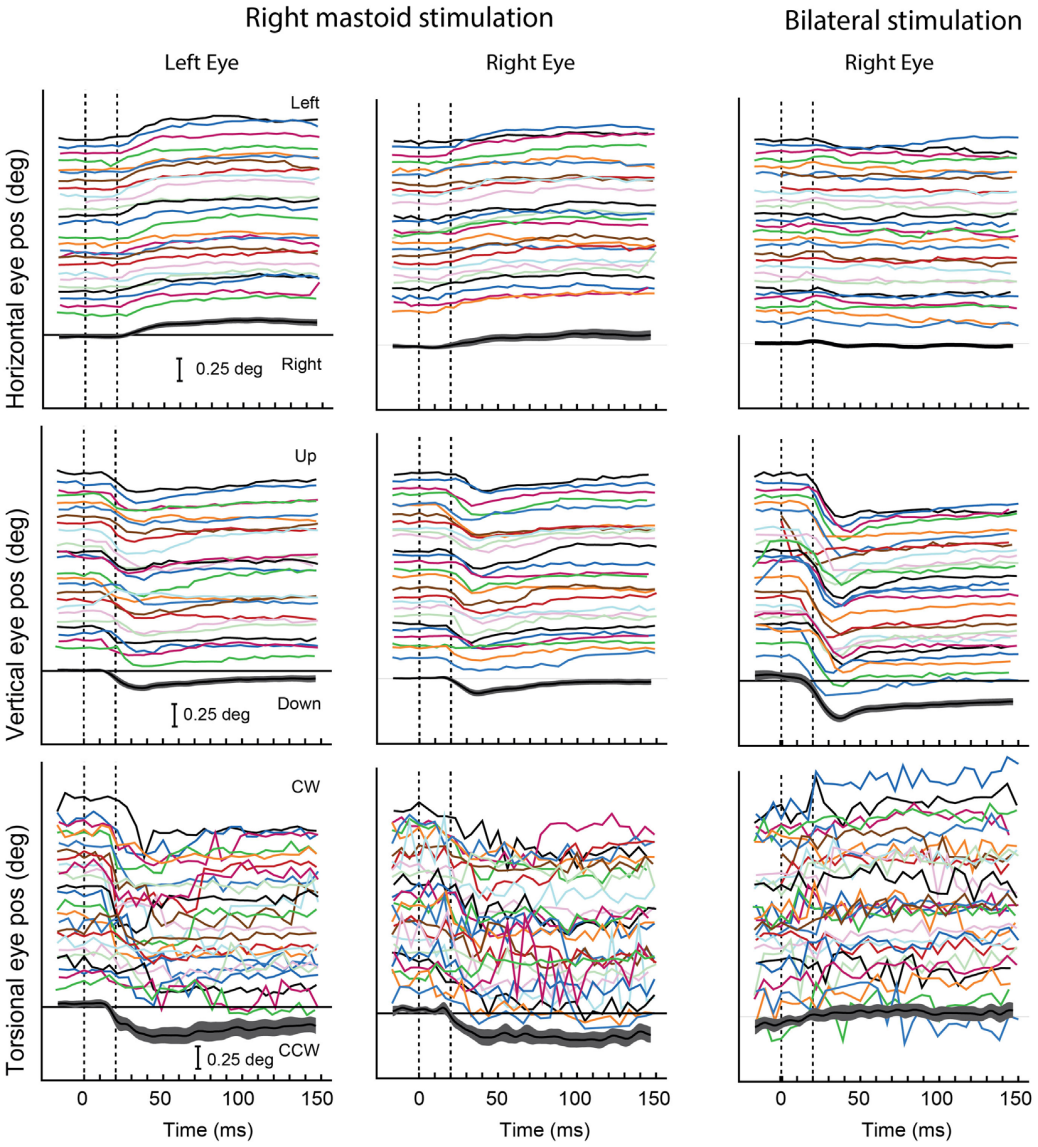
**Video recordings of horizontal, vertical, and torsional eye movements recorded simultaneously from the left and right eye in one subject during right mastoid stimulation (columns 1 and 2) and bilateral mastoid stimulation with a B71 bone oscillator**. Tone bursts of 20 ms were presented at 360-ms intervals over a 12-s recording period. The bottom trace in each panel shows the mean values and 95% confidence intervals of the individual responses in that panel. The eye moves horizontally and torsionally away from the side of mastoid stimulation during unilateral mastoid stimulation, but there is no clear response in either horizontal or torsion during simultaneous bilateral stimulation. The eye moves vertically down during both unilateral and bilateral mastoid stimulation, the response being greater during bilateral than unilateral stimulation. Reprinted from Ref. (138), © New York Academy of Sciences, 2011.

There are profound differences in otolith-mediated responses—even eye-movement responses—due to the characteristics of the otolithic stimuli. For example: on the one hand are maintained eye-movement responses to maintained otolithic stimuli, such as maintained roll-tilt. On the other hand, there are brief eye-movement responses to transient otolithic stimuli such as clicks or brief tone bursts (Figure 6). The otoliths transduce both of these very different stimulus aspects, and we suggest they do so by means of the different receptor types and the different sustained and transient neural systems. We discuss these examples below.

A maintained rolled head position alters the linear acceleration stimulus to the otoliths and causes both eyes to adopt a rolled position in the orbit in the opposite direction to head tilt (82–84). This response is called ocular counterrolling (OCR). It is a maintained ocular response with very little adaptation, even over minutes, to the maintained otolithic stimulus (85). OCR would appear to be due to the action of regular afferents which show very little adaptation to maintained otolith stimulation (7) in contrast to irregular afferents. These regular afferents are bouton or dimorphic afferents and receive input primarily from extrastriolar receptors, many of them being type II receptors (86), so we suggest that OCR reflects primarily the activity of the sustained system—regular otolithic afferents.

In contrast, brief bursts of 500-Hz BCV, which physiology has shown to selectively activate otolithic irregular afferents originating from striolar receptors, cause short-latency stimulus-locked eye movements in guinea pigs (87) and also in humans (see Figure 6) (88, 89). The pattern of these vibration-induced eye movements is consistent with the pattern of cat eye movements to direct electrical stimulation of the utricular nerve (90). We suggest these small vibration-induced eye movements reflect the action of the transient system, since neurobiotin staining has shown that it is irregular afferents synapsing on striolar type I receptors which are differentially activated by the same 500-Hz vibration stimulus eliciting the eye movements. In confirmation is the fact that the analogous guinea pig eye-movement response to 500 Hz BCV is abolished by ITG at low concentration which does not affect auditory brainstem threshold response (87).

It has been argued that irregular neurons comprise the transient system. But how is it that their activation, for example, by sustained vibration, can result in maintained nystagmus? For example, maintained 100-Hz vibration stimulation of one mastoid in a patient with unilateral loss induces a nystagmus during the stimulation (vibration-induced nystagmus). It would appear that such a maintained nystagmus during a maintained vibration stimulus should be due to the sustained system (regular neurons), which the physiology shows, are not activated by vibration.

The answer lies in the fact that for irregular neurons every single cycle of the stimulus is the direct stimulus to the receptor/afferent neuron. So a maintained 100-Hz vibration will cause 100 repetitive activations, once per cycle, of irregular afferents. So the average firing rate of irregular neurons will increase, just as would occur during a sustained deflection of the cupula during an angular acceleration, and a sustained nystagmus will result.

There are three pieces of evidence in support of such an account:

1. stimulus onset and offset. Vibration-induced activation of irregular neurons starts on the first cycle of the stimulus and so one would expect any nystagmus to start at the very onset of the vibration. That is the observed result [summarized in Dumas et al. (35)].
2. Similarly, the vibration-induced neural activation ceases abruptly at vibration offset and so one expects the nystagmus to cease abruptly without any decay or overshoot. That is the observed result (35).
3. As vibration frequency is increased on successive trials from 30 to 100 Hz, the number of phase-locked spikes/s will increase and so one expects nystagmus velocity to increase in a corresponding fashion. That is the observed result (35). For these reasons, we consider that a repetitive activation of the transient system can produce a maintained eye movement, or nystagmus, just as can a maintained deflection of the cupula during a long-duration angular acceleration. The train of action potentials in the afferent fibers will be indistinguishable for these two stimuli.

**Figure 7:**
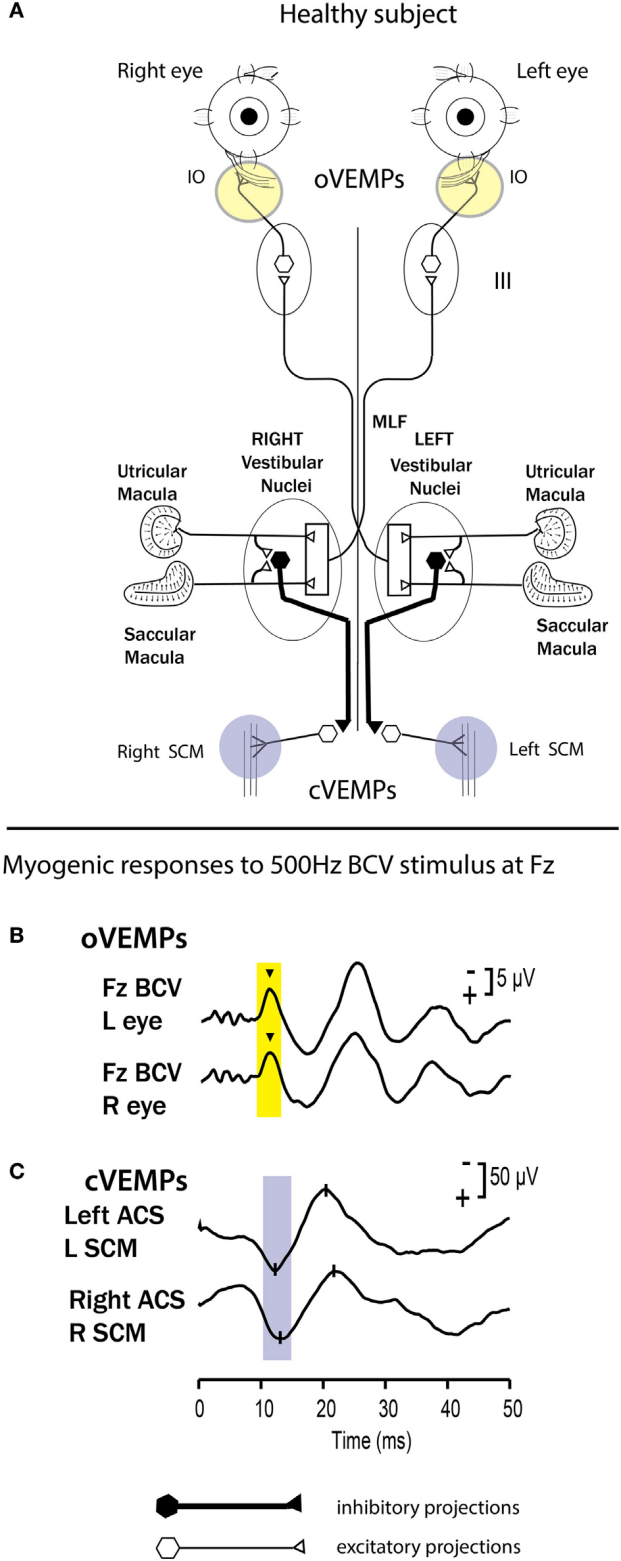
**(A)** A simplified schematic version of some of the known neural vestibulo-ocular and vestibulo-collic projections which underlie the ocular vestibular-evoked myogenic potential (oVEMP) and cervical vestibular-evoked myogenic potential (cVEMP) responses. It is based on known anatomical projections and physiological results from Suzuki et al. (90) that high-frequency electrical stimulation of the utricular nerve results in the activation of the contralateral inferior oblique (IO) and the ipsilateral superior oblique. Electrodes over the IO (circles) record the oVEMP. In human subjects, surface electrodes over the IO record the oVEMP, and the n10 component is shown by the inverted caret **(B)**. Fz bone-conducted vibration (BCV) refers to the fact that the BCV stimulus was delivered to the midline of the forehead at the hairline, and this location is known as Fz. Afferents from the saccular and utricular macula project to the vestibular nuclei, but the exact termination of these afferents in the vestibular nuclei is not presently known, so this figure represents the present uncertainty as an open box. The otolithic projections to other eye muscles are not shown. The projections of the saccular macula in the inferior vestibular nerve, synapsing on an inhibitory neuron in the vestibular nucleus (black hexagon), projecting to spinal motoneurons controlling the sternocleidomastoid muscle (SCM), are established by Uchino and Kushiro (139). Electrodes over contracted SCM muscles record the cVEMP. **(B,C)** Examples of averaged EMG responses, oVEMP responses **(B)** and cVEMP responses **(C)** for a healthy subject. The yellow and blue vertical bands mark the time of healthy oVEMP n10 and cVEMP p13 responses, respectively. This healthy subject displays n10 responses of similar magnitude and symmetrical p13-n23 responses. Reprinted from Ref. (138), © New York Academy of Sciences, 2011.

These results are complemented by other evidence from the preparatory potentials of the muscles activated by BCV, called VEMPs. These are recorded in response to brief clicks or bursts of 500-Hz ACS or BCV, which the physiological evidence shows to selectively activate otolith irregular neurons. In response to stimuli which are specific for otolith irregular afferents, these VEMPs are small EMGs recorded by electrodes beneath the eyes [ocular vestibular-evoked myogenic potential (oVEMPs)] or over the tensed sternocleidomastoid muscle [cervical vestibular-evoked myogenic potential (cVEMP)] (91–93) (Figure 7). The short latency potentials have been demonstrated to be indicators of utricular function (oVEMP) and saccular function (cVEMP) (87, 94, 95). VEMPs are now widely used clinically (71, 95).

So, in our terms, ocular counter roll is a test of static (maintained) otolith function—how the otoliths respond to maintained roll-tilt stimulus—in contrast to VEMP responses, which are tests of otolith dynamic responses to transient otolithic stimulation mediated by otolith striolar type I receptors and otolith irregular afferents. Regular afferents are not activated by these transient sounds and vibrations, which are such effective stimuli in eliciting otolith dynamic responses.

What is the clinical value of making this distinction? Immediately after surgical unilateral vestibular loss, there is asymmetrical OCR (82), but over time the effect of unilateral loss on OCR is not consistent, so that the OCR test of sustained function does not clearly detect a unilateral vestibular loss acutely and chronically. Presumably, processes of vestibular compensation are acting to reduce any asymmetry. The data from studies of the OCR of patients after vestibular neuritis also show great variability, but here it is not known conclusively whether otolith function may have returned after the neuritis (96, 97). On the other hand, VEMPs do show a clear asymmetry after unilateral vestibular loss acutely and chronically after surgical unilateral loss (98, 99) and after neuritis (100). The asymmetrical VEMPs are preserved for many years and probably permanently after the vestibular loss (92, 101–103). The clinical value lies in realizing that one cannot rely on the results of one of the tests of otolith function: the test of static otolithic function—OCR—which may show symmetrical responses indicating normal otolithic function, whereas in the same patient, the VEMPs may show the loss of transient otolithic function and so identify the patient’s problem (95, 104).

### Semicircular Canal Responses

During normal head movements, the neural drives from the sustained and transient systems are combined to generate smooth compensatory responses. For example, in response to brief, passive unpredictable horizontal head angular accelerations (a head impulse), they combine to generate a smooth compensatory eye movement—the vestibulo-ocular reflex (VOR). One index of the adequacy of vestibular responses is VOR gain, typically defined as the ratio of the area under the eye velocity record to the corresponding area under the head velocity record during the head impulse. This clinical test is called the video head impulse test (vHIT) (105, 106). VOR gain measured in this way is a global measure of the whole response to a head turn, and as such, it cannot show the fine detail of the time series of the response which may reflect the way in which the sustained and transient systems are combined. Fortuitously, an answer about how these systems work may be provided by the therapeutic treatment of patients with Meniere’s disease by the use of intratympanic injections of gentamicin (ITG) (107–111).

As noted above, histological studies in animals have shown that gentamicin differentially attacks type I receptors, and so ITG presumably selectively attenuates the contribution of type I receptors to generate responses to transient stimuli (79–81). We have argued that this loss of type I receptors will differentially degrade the irregular (transient) afferent system. Consistent with this account is the evidence that the human transient eye-movement response to brief 100-ms square wave pulses of electrical stimulation is impaired after systemic gentamicin, to a greater extent than the tonic response (112). The new evidence showing that galvanic stimulation acts on receptors (69) would predict such a decrease in the GVS response if the gentamicin-vulnerable type I receptors were degraded.

Recently, this ITG procedure for the treatment of patients with Meniere’s disease has been combined with sequential vHIT testing of semicircular canal function (106) as a monitor of the effect of ITG (110). In many patients, the pre-ITG treatment eye-movement response to impulsive head rotations may be normal, but after just a single dose, there is frequently a change in the exact time series of the eye-movement response. Most clinicians have been interested in the global VOR gain measure since it appears that even a small reduction in VOR gain after ITG is associated with a decrease in patient reports of vertigo (110). The small reduction in VOR gain is objective evidence that the ITG treatment has worked, and the ITG injections should stop in order to minimize risk of potential hearing loss. On the evidence presented here, we suggest the exact time series of the response should be examined in detail. From a physiological viewpoint, there is good reason for such a change in the response pattern because it is likely that the early response is dominated by the very fast dynamics of the type I receptors and irregular afferents for the semicircular canals (113, 114).

### Sustained and Transient Systems in Blood Pressure Control and Vestibulo-Sympathetic Responses?

There is now strong evidence that otolithic inputs contribute to early transient adjustments to human blood pressure control and muscle sympathetic nerve activity (MSNA) (115–122). The neural pathways for these functions are even being mapped (123–125). These responses are modulated by sinusoidal galvanic stimuli which preferentially activate irregular otolithic neurons, although regular neurons are activated by galvanic stimuli at high intensities (11). Given that BCV is such an effective stimulus for the transient system, it will be of interest to see if bursts of 500-Hz BCV also modulate blood pressure and MSNA since that would establish the transient circuit from otolithic striolar receptors to sympathetic responses.

### Escape Movements

Recent evidence suggests that type I receptors may even have a role in triggering escape behavior in response to hypoxia, and by implication in sudden infant death syndrome. Under hypoxic conditions, healthy mice show marked escape movements, but following ITG (which most probably disables the transient system selectively)—mice in the same hypoxic conditions show little escape movement (126–128). The neural pathways by which the transient system triggers escape behavior is not at all clear, but the possible significance of these results demands replication.

### Proportional–Integral–Derivative (PID) Control

The existence of these complementary transient and sustained systems seems to reflect a fundamental principle in the design of robotic control of limb movement. Designers of robotics have adopted a principle for initiation of movement of robots called a PID controller (129, 130). The principle is that movement controllers should be governed not by a single control signal, such as velocity, but by a combination of acceleration, velocity, and position signals (131). Controlling movement by just one signal (e.g., velocity) does not ensure dynamical stability.

There is a big advantage in such a combination especially at the onset of a movement, since the acceleration component initiates the responses rapidly. If there had only been a velocity signal at the onset, the early response would have been inadequate. The appropriate combination of acceleration and velocity provides a functionally effective way of dealing with response initiation. In an analogous fashion, we suggest these transient and sustained inputs are combined to yield dynamically stable oculomotor responses (and postural responses) analogous to PID control. Zhou et al. (132) have shown how a PID model can closely approximate oculomotor performance.

### A Complementary View

A series of studies from Cullen’s group has approached the regular–irregular distinction from the point of view of analyzing the neural coding of information about self motion. Their analysis of the responses of regular and irregular monkey single neurons to repetitions of various vestibular stimuli leads to the conclusion that it is irregular neurons that have distinct advantages in self motion coding, for example, great temporal precision. Regular afferents code information *via* changes in firing rate, whereas irregular afferents give information about self motion *via* precise spike timing (133–137). The evidence we have presented here, such as the phase locking of irregular neurons to very high vibration frequencies, is exactly consistent with such ideas.

## Summary

The old ideas of the otoliths being uniform structures functioning mainly to signal direction of gravity or low-frequency linear acceleration is just totally inadequate. It is now being replaced by evidence showing that there is complex anatomical differentiation of receptors and afferents in each macula and also in the cristae of the canals. This anatomical diversity is matched by afferent diversity of physiological response with different neurons signaling sustained aspects of the stimulus as opposed to other neurons signaling changes in stimulation (the transient system). There is corresponding differentiation of neural types and neural responses at the vestibular nuclei. These different systems—sustained and transient—are differentially susceptible to ototoxic antibiotics, have different frequency responses, different sensitivity to galvanic stimulation, and most interestingly differential sensitivity to high-frequency BCV (500 Hz) and ACS. These differential sensitivities are now allowing the differential contribution of sustained and transient systems to vestibular responses, such as eye movements, and possibly other vestibular-evoked responses—such as blood pressure—as well. The prospect of particular treatments targeting one or the other of the systems is now being realized in the clinic by therapeutic treatment of ITG preferentially attacking type I receptors with reported benefit to patients while retaining considerable vestibular function. This paper has highlighted parallels between the response characteristics of vestibular afferents and basic principles of robotic design.

The old terms “vestibular stimulation” or “otolith stimulation” are just too vague—we now know that afferent input with very different dynamics conveys a range of different information. The evidence reviewed in this paper has pointed to the value of regarding vestibular responses in terms of the transient and sustained characteristics.

## Dedication

This review is dedicated to Bernard Cohen who has made so many pioneering contributions to understanding vestibular function.

## Author Contributions

IC wrote the paper. P-PV contributed the section about central vestibular responses, HM contributed to the section on PID, and CW contributed to the section about clinical testing. All authors reviewed the text and approved the final paper.

## Conflict of Interest Statement

IC and HM are unpaid consultants to GN Otometrics, Taastrup, Denmark, but have received support from GN Otometrics for travel and attendance at conferences and workshops. For all other authors, the research was conducted in the absence of any commercial or financial relationships that could be construed as a potential conflict of interest.

## Abbreviations

ABR, auditory brainstem response; ACS, air-conducted sound; BCV, bone-conducted vibration; Fz, the midline of forehead at the hairline; GVS, galvanic vestibular stimulation; IO, inferior oblique eye muscle; ITG, intratympanic gentamicin; MSNA, muscle sympathetic nerve activity; VEMP, vestibular-evoked myogenic potential; cVEMP, cervical vestibular-evoked myogenic potential; oVEMP, ocular vestibular-evoked myogenic potential; OCR, ocular counterrolling; PID, proportional–integral–derivative; SCD, semicircular canal dehiscence; n10, the initial negative potential of the oVEMP at about 10 ms latency; SCM, sternocleidomastoid muscle; VIN, vibration-induced nystagmus; VsEP, vestibular-evoked potential.

## References

[1] Precht W Shimazu H Functional connections of tonic and kinetic vestibular neurons with primary vestibular afferents. J Neurophysiol (1965) 28(6):1014–28. 5295929 10.1152/jn.1965.28.6.1014

[2] Shimazu H Precht W Tonic and kinetic responses of cats vestibular neurons to horizontal angular acceleration. J Neurophysiol (1965) 28(6):991–1013. 5295930 10.1152/jn.1965.28.6.991

[3] Shimazu H Precht W Inhibition of central vestibular neurons from contralateral labyrinth and its mediating pathway. J Neurophysiol (1966) 29(3):467–92. 5961161 10.1152/jn.1966.29.3.467

[4] Fernandez C Goldberg JM Physiology of peripheral neurons innervating semicircular canals of the squirrel monkey. II. Response to sinusoidal stimulation and dynamics of peripheral vestibular system. J Neurophysiol (1971) 34(4):661–75. 5000363 10.1152/jn.1971.34.4.661

[5] Goldberg JM Fernandez C Physiology of peripheral neurons innervating semicircular canals of the squirrel monkey. 3. Variations among units in their discharge properties. J Neurophysiol (1971) 34(4):676–84. 5000364 10.1152/jn.1971.34.4.676

[6] Goldberg JM Fernandez C Physiology of peripheral neurons innervating semicircular canals of the squirrel monkey. I. Resting discharge and response to constant angular accelerations. J Neurophysiol (1971) 34(4):635–60. 5000362 10.1152/jn.1971.34.4.635

[7] Fernandez C Goldberg JM Physiology of peripheral neurons innervating otolith organs of the squirrel monkey. I. Response to static tilts and to long-duration centrifugal force. J Neurophysiol (1976) 39(5):970–84. 824412 10.1152/jn.1976.39.5.970

[8] Fernandez C Goldberg JM Physiology of peripheral neurons innervating otolith organs of the squirrel monkey. II. Directional selectivity and force-response relations. J Neurophysiol (1976) 39(5):985–95. 824413 10.1152/jn.1976.39.5.985

[9] Fernandez C Goldberg JM Physiology of peripheral neurons innervating otolith organs of the squirrel monkey. III. Response dynamics. J Neurophysiol (1976) 39(5):996–1008. 824414 10.1152/jn.1976.39.5.996

[10] Goldberg JM. Afferent diversity and the organization of central vestibular pathways. Exp Brain Res (2000) 130(3):277–97. 10.1007/s002210050033 10706428 PMC3731078

[11] Kim J Curthoys IS. Responses of primary vestibular neurons to galvanic vestibular stimulation (GVS) in the anaesthetised guinea pig. Brain Res Bull (2004) 64(3):265–71. 10.1016/j.brainresbull.2004.07.008 15464864

[12] Goldberg JM Smith CE Fernandez C. Relation between discharge regularity and responses to externally applied galvanic currents in vestibular nerve afferents of the squirrel monkey. J Neurophysiol (1984) 51(6):1236–56. 6737029 10.1152/jn.1984.51.6.1236

[13] McCue MP Guinan JJ Jr. Acoustically responsive fibers in the vestibular nerve of the cat. J Neurosci (1994) 14(10):6058–70. 7931562 10.1523/JNEUROSCI.14-10-06058.1994 PMC6576982

[14] Murofushi T Curthoys IS Topple AN Colebatch JG Halmagyi GM. Responses of guinea pig primary vestibular neurons to clicks. Exp Brain Res (1995) 103(1):174–8. 10.1007/BF00241975 7615033

[15] Young ED Fernandez C Goldberg JM. Responses of squirrel monkey vestibular neurons to audio-frequency sound and head vibration. Acta Otolaryngol (1977) 84(5–6):352–60. 10.3109/00016487709123977 303426

[16] Curthoys IS Peripheral vestibular responses to sound. Neuroembryol Aging (2004) 3(4):207–14. 10.1159/000096798

[17] Curthoys IS The new vestibular stimuli: sound and vibration. Anatomical, physiological and clinical evidence. Exp Brain Res (2017) 235(4):957–72. 10.1007/s00221-017-4874-y 28130556

[18] Curthoys IS Kim J McPhedran SK Camp AJ. Bone conducted vibration selectively activates irregular primary otolithic vestibular neurons in the guinea pig. Exp Brain Res (2006) 175(2):256–67. 10.1007/s00221-006-0544-1 16761136

[19] Curthoys IS Vulovic V. Vestibular primary afferent responses to sound and vibration in the guinea pig. Exp Brain Res (2011) 210(3–4):347–52. 10.1007/s00221-010-2499-5 21113779

[20] Curthoys IS Vulovic V Burgess AM Sokolic L Goonetilleke SC The response of guinea pig primary utricular and saccular irregular neurons to bone-conducted vibration (BCV) and air-conducted, sound (ACS). Hear Res (2016) 331:131–43. 10.1016/j.heares.2015.10.019 26626360

[21] Curthoys IS Vulovic V Sokolic L Pogson J Burgess AM. Irregular primary otolith afferents from the guinea pig utricular and saccular maculae respond to both bone conducted vibration and to air conducted sound. Brain Res Bull (2012) 89(1–2):16–21. 10.1016/j.brainresbull.2012.07.007 22814095

[22] Rose JE Brugge JF Anderson DJ Hind JE Phase-locked response to low-frequency tones in single auditory nerve fibers of the squirrel monkey. J Neurophysiol (1967) 30(4):769–93. 4962851 10.1152/jn.1967.30.4.769

[23] McCue MP Guinan JJ Jr. Spontaneous activity and frequency selectivity of acoustically responsive vestibular afferents in the cat. J Neurophysiol (1995) 74(4):1563–72. 8989393 10.1152/jn.1995.74.4.1563

[24] McCue MP Guinan JJ Jr. Sound-evoked activity in primary afferent neurons of a mammalian vestibular system. Am J Otol (1997) 18(3):355–60. 9149831

[25] Murofushi T Curthoys IS. Physiological and anatomical study of click-sensitive primary vestibular afferents in the guinea pig. Acta Otolaryngol (1997) 117(1):66–72. 10.3109/00016489709117994 9039484

[26] Murofushi T Curthoys IS Gilchrist DP. Response of guinea pig vestibular nucleus neurons to clicks. Exp Brain Res (1996) 111(1):149–52. 10.1007/BF00229565 8891646

[27] Zhu H Tang X Wei W Maklad A Mustain W Rabbitt R Input-output functions of vestibular afferent responses to air-conducted clicks in rats. J Assoc Res Otolaryngol (2014) 15(1):73–86. 10.1007/s10162-013-0428-6 24297262 PMC3901862

[28] Zhu H Tang X Wei W Mustain W Xu Y Zhou W. Click-evoked responses in vestibular afferents in rats. J Neurophysiol (2011) 106(2):754–63. 10.1152/jn.00003.2011 21613592

[29] Iwasaki S Chihara Y Komuta Y Ito K Sahara Y. Low-voltage-activated potassium channels underlie the regulation of intrinsic firing properties of rat vestibular ganglion cells. J Neurophysiol (2008) 100(4):2192–204. 10.1152/jn.01240.2007 18632889

[30] Kalluri R Xue J Eatock RA. Ion channels set spike timing regularity of mammalian vestibular afferent neurons. J Neurophysiol (2010) 104(4):2034–51. 10.1152/jn.00396.2010 20660422 PMC2957450

[31] Liu XP Wooltorton JRA Gaboyard-Niay S Yang FC Lysakowski A Eatock RA Sodium channel diversity in the vestibular ganglion: Na(V)1.5, Na(V)1.8, and tetrodotoxin-sensitive currents. J Neurophysiol (2016) 115(5):2536–55. 10.1152/jn.00902.2015 26936982 PMC4922472

[32] Carey JP Hirvonen TP Hullar TE Minor LB. Acoustic responses of vestibular afferents in a model of superior canal dehiscence. Otol Neurotol (2004) 25(3):345–52. 10.1097/00129492-200405000-00024 15129116

[33] Curthoys IS Grant JW. How does high-frequency sound or vibration activate vestibular receptors? Exp Brain Res (2015) 233(3):691–9. 10.1007/s00221-014-4192-6 25567092

[34] Curthoys IS Vulovic V Pogson J Sokolic L Responses of Guinea Pig Primary Vestibular Afferents to Low Frequency (50-100Hz) Bone Conducted Vibration (BCV) – The Neural Basis of Vibration Induced Nystagmus. Program No 57406 2012 Neuroscience Meeting Planner. New Orleans, LA: Society for Neuroscience (2013).

[35] Dumas G Curthoys IS Lion A Perrin A Schmerber S The skull vibration induced nystagmus test (SVINT) of vestibular function – a review. Front Neurol (2017) 8:41 10.3389/fneur.2017.00041 28337171 PMC5343042

[36] Dumas G Perrin P Schmerber S Lavieille JP Nystagmus and vibration test research of mechanisms, theoretical methods: on 52 cases of unilateral vestibular lesions. Rev Laryngol Otol Rhinol (2003) 124(2):75–83. 14564821

[37] Watanuki K Meyer A Morphological study of sensory epithelium of vestibular organs. Tohoku J Exp Med (1971) 104(1):55–63. 10.1620/tjem.104.55 5314824

[38] Lim DJ Morphological and physiological correlates in cochlear and vestibular sensory epithelia. Scan Electron Microsc (1976) 2:269–76.

[39] Hunter-Duvar IM An electron-microscopic study of the vestibular sensory epithelium. Acta Otolaryngol (1983) 95(5–6):494–507. 10.3109/00016488309139434 6880659

[40] Brichta AM Peterson EH. Functional architecture of vestibular primary afferents from the posterior semicircular canal of a turtle, *Pseudemys (Trachemys) scripta elegans*. J Comp Neurol (1994) 344(4):481–507. 10.1002/cne.903440402 7929889

[41] Kessel RG Kardon RH The shape, polarization, and innervation of sensory hair cells in the guinea pig crista ampullaris and macula utriculi. Scan Electron Microsc (1979) 3:962–74. 42972

[42] Lim DJ Fine morphology of the otoconial membrane and its relationship to the sensory epithelium. Scan Electron Microsc (1979) 3:929–38. 524064

[43] Lindeman HH Studies on the morphology of the sensory regions of the vestibular apparatus with 45 figures. Ergeb Anat Entwicklungsgesch (1969) 42(1):1–113. 5310109

[44] Spoon C Grant W. Biomechanics of hair cell kinocilia: experimental measurement of kinocilium shaft stiffness and base rotational stiffness with Euler-Bernoulli and Timoshenko beam analysis. J Exp Biol (2011) 214(Pt 5):862–70. 10.1242/jeb.051151 21307074 PMC3036549

[45] Dunlap MD Grant JW. Experimental measurement of utricle system dynamic response to inertial stimulus. J Assoc Res Otolaryngol (2014) 15(4):511–28. 10.1007/s10162-014-0456-x 24845403 PMC4141440

[46] Grant W Best W Otolith-organ mechanics – lumped parameter model and dynamic-response. Aviat Space Environ Med (1987) 58(10):970–6. 3314853

[47] Songer JE Eatock RA. Tuning and timing in mammalian type I hair cells and calyceal synapses. J Neurosci (2013) 33(8):3706–24. 10.1523/jneurosci.4067-12.2013 23426697 PMC3857958

[48] Geleoc GSG Lennan GWT Richardson GP Kros CJ. A quantitative comparison of mechanoelectrical transduction in vestibular and auditory hair cells of neonatal mice. Proc Biol Sci (1997) 264(1381):611–21. 10.1098/rspb.1997.0087 9149428 PMC1688386

[49] Spoon C Moravec WJ Rowe MH Grant JW Peterson EH. Steady-state stiffness of utricular hair cells depends on macular location and hair bundle structure. J Neurophysiol (2011) 106(6):2950–63. 10.1152/jn.00469.2011 21918003 PMC3234090

[50] Songer JE Rosowski JJ. The effect of superior canal dehiscence on cochlear potential in response to air-conducted stimuli in chinchilla. Hear Res (2005) 210(1–2):53–62. 10.1016/j.heares.2005.07.003 16150562 PMC1513126

[51] Songer JE Rosowski JJ. The effect of superior-canal opening on middle-ear input admittance and air-conducted stapes velocity in chinchilla. J Acoust Soc Am (2006) 120(1):258–69. 10.1121/1.2204356 16875223 PMC2726575

[52] Boyle R Highstein SM Resting discharge and response dynamics of horizontal semicircular canal afferents of the toadfish, opsanus-tau. J Neurosci (1990) 10(5):1557–69. 2332797 10.1523/JNEUROSCI.10-05-01557.1990 PMC6570080

[53] Fontilla MF Peterson EH. Kinocilia heights on utricular hair cells. Hear Res (2000) 145(1–2):8–16. 10.1016/S0378-5955(00)00068-X 10867272

[54] Li A Xue J Peterson EH. Architecture of the mouse utricle: macular organization and hair bundle heights. J Neurophysiol (2008) 99(2):718–33. 10.1152/jn.00831.2007 18046005

[55] Xue JB Peterson EH. Hair bundle heights in the utricle: differences between macular locations and hair cell types. J Neurophysiol (2006) 95(1):171–86. 10.1152/jn.00800.2005 16177175

[56] Boyle R Goldberg JM Highstein SM. Inputs from regularly and irregularly discharging vestibular nerve afferents to secondary neurons in squirrel monkey vestibular nuclei. III. Correlation with vestibulospinal and vestibuloocular output pathways. J Neurophysiol (1992) 68(2):471–84. 1527570 10.1152/jn.1992.68.2.471

[57] Chen-Huang C McCrea RA Goldberg JM. Contributions of regularly and irregularly discharging vestibular-nerve inputs to the discharge of central vestibular neurons in the alert squirrel monkey. Exp Brain Res (1997) 114(3):405–22. 10.1007/pl00005650 9187277

[58] Highstein SM Goldberg JM Moschovakis AK Fernandez C. Inputs from regularly and irregularly discharging vestibular nerve afferents to secondary neurons in the vestibular nuclei of the squirrel monkey. II. Correlation with output pathways of secondary neurons. J Neurophysiol (1987) 58(4):719–38. 2445938 10.1152/jn.1987.58.4.719

[59] Eugene D Idoux E Beraneck M Moore LE Vidal PP. Intrinsic membrane properties of central vestibular neurons in rodents. Exp Brain Res (2011) 210(3–4):423–36. 10.1007/s00221-011-2569-3 21331527

[60] Beraneck M Pfanzelt S Vassias I Rohregger M Vibert N Vidal PP Differential intrinsic response dynamics determine synaptic signal processing in frog vestibular neurons. J Neurosci (2007) 27(16):4283–96. 10.1523/jneurosci.5232-06.2007 17442812 PMC6672329

[61] Beraneck M Straka H. Vestibular signal processing by separate sets of neuronal filters. J Vestib Res (2011) 21(1):5–19. 10.3233/ves-2011-0396 21422539

[62] Pfanzelt S Roessert C Rohregger M Glasauer S Moore LE Straka H. Differential dynamic processing of afferent signals in frog tonic and phasic second-order vestibular neurons. J Neurosci (2008) 28(41):10349–62. 10.1523/jneurosci.3368-08.2008 18842894 PMC6671017

[63] Rossert C Straka H Glasauer S Moore LE. Frequency-domain analysis of intrinsic neuronal properties using high-resistant electrodes. Front Neurosci (2009) 3:64. 10.3389/neuro.17.002.2009 20582288 PMC2858610

[64] Straka H Beraneck M Rohregger M Moore LE Vidal PP Vibert N. Second-order vestibular neurons form separate populations with different membrane and discharge properties. J Neurophysiol (2004) 92(2):845–61. 10.1152/jn.00107.2004 15044516

[65] Straka H Lambert FM Pfanzelt S Beraneck M Vestibulo-ocular signal transformation in frequency-tuned channels. In: Strupp M Buttner U Cohen B, editors. Basic and Clinical Aspects of Vertigo and Dizziness. (Vol. 1164). New York: The New York Academy of Sciences (2009). p. 37–44. 10.1111/j.1749-6632.2008.03740.x 19645878

[66] Straka H Vibert N Vidal PP Moore LE Dutia MB Intrinsic membrane properties of vertebrate vestibular neurons: function, development and plasticity. Prog Neurobiol (2005) 76(6):349–92. 10.1016/j.pneurobio.2005.10.002 16263204

[67] Beraneck M Cullen KE. Activity of vestibular nuclei neurons during vestibular and optokinetic stimulation in the alert mouse. J Neurophysiol (2007) 98(3):1549–65. 10.1152/jn.00590.2007 17625061

[68] Babalian AL Vidal PP. Floccular modulation of vestibuloocular pathways and cerebellum-related plasticity: an in vitro whole brain study. J Neurophysiol (2000) 84(5):2514–28. 11067994 10.1152/jn.2000.84.5.2514

[69] Gensberger KD Kaufmann AK Dietrich H Branoner F Banchi R Chagnaud BP Galvanic vestibular stimulation: cellular substrates and response patterns of neurons in the vestibulo-ocular network. J Neurosci (2016) 36(35):9097–110. 10.1523/jneurosci.4239-15.2016 27581452 PMC6601907

[70] Rosengren SM Jombik P Halmagyi GM Colebatch JG. Galvanic ocular vestibular evoked myogenic potentials provide new insight into vestibulo-ocular reflexes and unilateral vestibular loss. Clin Neurophysiol (2009) 120(3):569–80. 10.1016/j.clinph.2008.12.001 19269890

[71] Weber KP Rosengren SM Clinical utility of ocular vestibular-evoked myogenic potentials (oVEMPs). Curr Neurol Neurosci Rep (2015) 15(5):22 10.1007/s11910-015-0548-y 25773001

[72] Brown D Electrovestibulography: a review of recording techniques, nomenclature, and new experimental findings. Front Neurotol (2017).

[73] Jones SM Erway LC Bergstrom RA Schimenti JC Jones TA. Vestibular responses to linear acceleration are absent in otoconia-deficient C57BL/6JEi-het mice. Hear Res (1999) 135(1–2):56–60. 10.1016/s0378-5955(99)00090-8 10491954

[74] Jones SM Jones TA. Ontogeny of vestibular compound action potentials in the domestic chicken. J Assoc Res Otolaryngol (2000) 1(3):232–42. 10.1007/s101620010026 11545229 PMC2504545

[75] Jones SM Jones TA Shukla R. Short latency vestibular evoked potentials in the Japanese quail (*Coturnix coturnix japonica*). J Comp Physiol A (1997) 180(6):631–8. 10.1007/s003590050079 9190045

[76] Jones TA Jones SM. Short latency compound action potentials from mammalian gravity receptor organs. Hear Res (1999) 136(1–2):75–85. 10.1016/s0378-5955(99)00110-0 10511626

[77] Chihara Y Wang V Brown DJ. Evidence for the utricular origin of the vestibular short-latency-evoked potential (VsEP) to bone-conducted vibration in guinea pig. Exp Brain Res (2013) 229(2):157–70. 10.1007/s00221-013-3602-5 23780310

[78] Jones TA Jones SM Vijayakumar S Brugeaud A Bothwell M Chabbert C. The adequate stimulus for mammalian linear vestibular evoked potentials (VsEPs). Hear Res (2011) 280(1–2):133–40. 10.1016/j.heares.2011.05.005 21664446 PMC3826178

[79] Hirvonen TP Minor LB Hullar TE Carey JP. Effects of intratympanic gentamicin on vestibular afferents and hair cells in the chinchilla. J Neurophysiol (2005) 93(2):643–55. 10.1152/jn.00160.2004 15456806

[80] Lue JH Day AS Cheng PW Young YH. Vestibular evoked myogenic potentials are heavily dependent on type I hair cell activity of the saccular macula in guinea pigs. Audiol Neurootol (2009) 14(1):59–66. 10.1159/000156701 18812694

[81] Lyford-Pike S Vogelheim C Chu E Della Santina CC Carey JP. Gentamicin is primarily localized in vestibular type I hair cells after intratympanic administration. J Assoc Res Otolaryngol (2007) 8(4):497–508. 10.1007/s10162-007-0093-8 17899270 PMC2538341

[82] Diamond SG Markham CH Binocular counterrolling in humans with unilateral labyrinthectomy and in normal controls. Ann N Y Acad Sci (1981) 374:69–79. 10.1111/j.1749-6632.1981.tb30861.x 6951454

[83] Diamond SG Markham CH. Ocular counterrolling as an indicator of vestibular otolith function. Neurology (1983) 33(11):1460–9. 10.1212/WNL.33.11.1460 6605496

[84] Diamond SG Markham CH Simpson NE Curthoys IS. Binocular counterrolling in humans during dynamic rotation. Acta Otolaryngol (1979) 87(5–6):490–8. 10.3109/00016487909126457 313656

[85] Moore ST Curthoys IS McCoy SG VTM – an image-processing system for measuring ocular torsion. Comput Methods Programs Biomed (1991) 35(3):219–30. 10.1016/0169-2607(91)90124-C 1935015

[86] Fernandez C Goldberg JM Baird RA. The vestibular nerve of the chinchilla. III. Peripheral innervation patterns in the utricular macula. J Neurophysiol (1990) 63(4):767–80. 2341875 10.1152/jn.1990.63.4.767

[87] Vulovic V Curthoys IS. Bone conducted vibration activates the vestibulo-ocular reflex in the guinea pig. Brain Res Bull (2011) 86(1–2):74–81. 10.1016/j.brainresbull.2011.06.013 21745548

[88] Cornell ED Burgess AM MacDougall HG Curthoys IS. Vertical and horizontal eye movement responses to unilateral and bilateral bone conducted vibration to the mastoid. J Vestib Res (2009) 19(1–2):41–7. 10.3233/VES-2009-0338 19893196

[89] Cornell ED Burgess AM MacDougall HG Curthoys IS. Bone conducted vibration to the mastoid produces horizontal, vertical and torsional eye movements. J Vestib Res (2015) 25(2):91–6. 10.3233/ves-150550 26410673

[90] Suzuki JI Tokumasu K Goto K Eye movements from single utricular nerve stimulation in the cat. Acta Otolaryngol (1969) 68(4):350–62. 10.3109/00016486909121573 5309166

[91] Colebatch JG Halmagyi GM Skuse NF. Myogenic potentials generated by a click-evoked vestibulocollic reflex. J Neurol Neurosurg Psychiatry (1994) 57(2):190–7. 10.1136/jnnp.57.2.190 8126503 PMC1072448

[92] Iwasaki S McGarvie LA Halmagyi GM Burgess AM Kim J Colebatch JG Head taps evoke a crossed vestibulo-ocular reflex. Neurology (2007) 68(15):1227–9. 10.1212/01.wnl.0000259064.80564.21 17420408

[93] Rosengren SM Todd NPM Colebatch JG. Vestibular-evoked extraocular potentials produced by stimulation with bone-conducted sound. Clin Neurophysiol (2005) 116(8):1938–48. 10.1016/j.clinph.2005.03.019 15979939

[94] Curthoys IS. A critical review of the neurophysiological evidence underlying clinical vestibular testing using sound, vibration and galvanic stimuli. Clin Neurophysiol (2010) 121(2):132–44. 10.1016/j.clinph.2009.09.027 19897412

[95] Curthoys IS. The interpretation of clinical tests of peripheral vestibular function. Laryngoscope (2012) 122(6):1342–52. 10.1002/lary.23258 22460150

[96] Schmid-Priscoveanu A Straumann D Bohmer A Obzina H. Vestibulo-ocular responses during static head roll and three-dimensional head impulses after vestibular neuritis. Acta Otolaryngol (1999) 119(7):750–7. 10.1080/00016489950180379 10687930

[97] Schmid-Priscoveanu A Straumann D Bohmer A Obzina H Is static ocular counterroll asymmetric after vestibular neuritis? In: Claussen CF Haid CT Hofferberth B, editors. Equilibrium Research, Clinical Equilibriometry and Modern Treatment. (Vol. 1201). Amsterdam: Elsevier (2000). p. 442–442.

[98] Chiarovano E Zamith F Vidal PP de Waele C. Ocular and cervical VEMPs: a study of 74 patients suffering from peripheral vestibular disorders. Clin Neurophysiol (2011) 122(8):1650–9. 10.1016/j.clinph.2011.01.006 21306945

[99] Kinoshita M Iwasaki S Fujimoto C Inoue A Egami N Chihara Y Ocular vestibular evoked myogenic potentials in response to air-conducted sound and bone-conducted vibration in vestibular schwannoma. Otol Neurotol (2013) 34(7):1342–8. 10.1097/MAO.0b013e31828d6539 23945552

[100] Manzari L Burgess AM MacDougall HG Curthoys IS. Vestibular function after vestibular neuritis. Int J Audiol (2013) 52(10):713–8. 10.3109/14992027.2013.809485 23902522

[101] Iwasaki S Murofushi T Chihara Y Ushio M Suzuki M Curthoys IS Ocular vestibular evoked myogenic potentials to bone-conducted vibration in vestibular schwannomas. Otol Neurotol (2010) 31(1):147–52. 10.1097/MAO.0b013e3181c0e670 19816224

[102] Iwasaki S Smulders YE Burgess AM McGarvie LA MacDougall HG Halmagyi GM Ocular vestibular evoked myogenic potentials in response to bone-conducted vibration of the midline forehead at Fz. A new indicator of unilateral otolithic loss. Audiol Neurootol (2008) 13(6):396–404. 10.1159/000148203 18663292

[103] Manzari L Tedesco A Burgess AM Curthoys IS. Ocular vestibular-evoked myogenic potentials to bone-conducted vibration in superior vestibular neuritis show utricular function. Otolaryngol Head Neck Surg (2010) 143(2):274–80. 10.1016/j.otohns.2010.03.020 20647134

[104] Chiarovano E Darlington C Vidal P-P Lamas G de Waele C. The role of cervical and ocular vestibular evoked myogenic potentials in the assessment of patients with vestibular schwannomas. PLoS One (2014) 9(8):e105026. 10.1371/journal.pone.0105026 25137289 PMC4138161

[105] MacDougall HG McGarvie LA Halmagyi GM Curthoys IS Weber KP. Application of the video head impulse test to detect vertical semicircular canal dysfunction. Otol Neurotol (2013) 34(6):974–9. 10.1097/MAO.0b013e31828d676d 23714711

[106] MacDougall HG Weber KP McGarvie LA Halmagyi GM Curthoys IS. The video head impulse test: diagnostic accuracy in peripheral vestibulopathy. Neurology (2009) 73(14):1134–41. 10.1212/WNL.0b013e3181bacf85 19805730 PMC2890997

[107] Bremer HG van Rooy I Pullens B Colijn C Stegeman I van der Zaag-Loonen HJ Intratympanic gentamicin treatment for Meniere’s disease: a randomized, double-blind, placebo-controlled trial on dose efficacy – results of a prematurely ended study. Trials (2014) 15:328 10.1186/1745-6215-15-328 25135244 PMC4141100

[108] Carey J. Intratympanic gentamicin for the treatment of Meniere’s disease and other forms of peripheral vertigo. Otolaryngol Clin North Am (2004) 37(5):1075–90. 10.1016/j.otc.2004.06.002 15474112

[109] Carey JP Vestibulotoxicity and management of vestibular disorders. Volta Rev (2005) 105(3):251–76.

[110] Marques P Manrique-Huarte R Perez-Fernandez N Single intratympanic gentamicin injection in Meniere’s disease: VOR change and prognostic usefulness. Laryngoscope (2015) 125(8):1915–20. 10.1002/lary.25156 25641686

[111] Viana LM Bahmad F Rauch SD. Intratympanic gentamicin as a treatment for drop attacks in patients with Meniere’s disease. Laryngoscope (2014) 124(9):2151–4. 10.1002/lary.24716 24729095

[112] Aw ST Todd MJ Aw GE Weber KP Halmagyi GM. Gentamicin vestibulotoxicity impairs human electrically evoked vestibulo-ocular reflex. Neurology (2008) 71(22):1776–82. 10.1212/01.wnl.0000335971.43443.d9 19029517

[113] Hullar TE Della Santina CC Hirvonen T Lasker DM Carey JP Minor LB. Responses of irregularly discharging chinchilla semicircular canal vestibular-nerve afferents during high-frequency head rotations. J Neurophysiol (2005) 93(5):2777–86. 10.1152/jn.01002.2004 15601735

[114] Kim KS Minor LB Della Santina CC Lasker DM. Variation in response dynamics of regular and irregular vestibular-nerve afferents during sinusoidal head rotations and currents in the chinchilla. Exp Brain Res (2011) 210(3–4):643–9. 10.1007/s00221-011-2600-8 21369854 PMC4010622

[115] Hammam E Bolton PS Kwok K Macefield VG Vestibular modulation of muscle sympathetic nerve activity during sinusoidal linear acceleration in supine humans. Front Neurosci (2014) 8:316 10.3389/fnins.2014.00316 25346657 PMC4191191

[116] Hammam E Hau CLV Wong KS Kwok K Macefield VG Vestibular modulation of muscle sympathetic nerve activity by the utricle during sub-perceptual sinusoidal linear acceleration in humans. Exp Brain Res (2014) 232(4):1379–88. 10.1007/s00221-014-3856-6 24504198

[117] Hammam E Kwok K Macefield VG. Modulation of muscle sympathetic nerve activity by low-frequency physiological activation of the vestibular utricle in awake humans. Exp Brain Res (2013) 230(1):137–42. 10.1007/s00221-013-3637-7 23852323

[118] Kaufmann H Biaggioni I Voustianiouk A Diedrich A Costa F Clarke R Vestibular control of sympathetic activity – an otolith-sympathetic reflex in humans. Exp Brain Res (2002) 143(4):463–9. 10.1007/s00221-002-1002-3 11914792

[119] Raphan T Cohen B Xiang YQ Yakushin SB A model of blood pressure, heart rate, and vaso-vagal responses produced by vestibulo-sympathetic activation. Front Neurosci (2016) 10:96 10.3389/fnins.2016.00096 27065779 PMC4814511

[120] Voustianiouk A Kaufmann H Diedrich A Raphan T Biaggioni I MacDougall H Electrical activation of the human vestibulo-sympathetic reflex. Exp Brain Res (2006) 171(2):251–61. 10.1007/s00221-005-0266-9 16308690

[121] Watenpaugh DE Cothron AV Wasmund SL Wasmund WL Carter R Muenter NK Do vestibular otolith organs participate in human orthostatic blood pressure control? Auton Neurosci (2002) 100(1–2):77–83. 10.1016/s1566-0702(02)00142-x 12422963

[122] Yates BJ Bolton PS Macefield VG. Vestibulo-sympathetic responses. Compr Physiol (2014) 4(2):851–87. 10.1002/cphy.c130041 24715571 PMC3999523

[123] Holstein GP Friedrich VL Martinelli GP Glutamate and GABA in vestibulo-sympathetic pathway neurons. Front Neuroanat (2016) 10:7 10.3389/fnana.2016.00007 26903817 PMC4744852

[124] Holstein GR Friedrich VL Martinelli GP. Projection neurons of the vestibulo-sympathetic reflex pathway. J Comp Neurol (2014) 522(9):2053–74. 10.1002/cne.23517 24323841 PMC3997612

[125] Holstein GR Friedrich VL Martinelli GP Imidazoleacetic acid-ribotide in vestibulo-sympathetic pathway neurons. Exp Brain Res (2016) 234(10):2747–60. 10.1007/s00221-016-4725-2 27411812 PMC5026920

[126] Allen T Garcia AJ Tang J Ramirez JM Rubens DD. Inner ear insult ablates the arousal response to hypoxia and hypercarbia. Neuroscience (2013) 253:283–91. 10.1016/j.neuroscience.2013.08.059 24021919 PMC3988803

[127] Allen T Juric-Sekhar G Campbell S Mussar KE Seidel K Tan J Inner ear insult suppresses the respiratory response to carbon dioxide. Neuroscience (2011) 175:262–72. 10.1016/j.neuroscience.2010.11.034 21130842

[128] Ramirez S Allen T Villagracia L Chae Y Ramirez JM Rubens DD. Inner ear lesion and the differential roles of hypoxia and hypercarbia in triggering active movements: potential implication for the sudden infant death syndrome. Neuroscience (2016) 337:9–16. 10.1016/j.neuroscience.2016.08.054 27634772

[129] Arimoto S Fundamental problems of robot control, Part I, Innovations in the realm of robot servo-loops. Robotica (1995) 13:19–27. 10.1017/S0263574700017446

[130] Su YX Zheng CH PID control for global finite-time regulation of robotic manipulators. Int J Syst Sci (2017) 48(3):547–58. 10.1080/00207721.2016.1193256

[131] Mergner T Maurer C Peterka RJ. A multisensory posture control model of human upright stance. Prog Brain Res (2003) 142:189–201. 10.1016/S0079-6123(03)42014-1 12693262

[132] Zhou Y Luo J Hu JQ Li HY Xie SR Bionic eye system based on fuzzy adaptive PID control. In IEEE Robotics and Automation Society, ROBIO 2012: 2012 IEEE International Conference on Robotics and Biomimetics Piscataway, NJ: IEEE (2012). p. 1268–72.

[133] Cullen KE. The neural encoding of self-motion. Curr Opin Neurobiol (2011) 21(4):587–95. 10.1016/j.conb.2011.05.022 21689924

[134] Cullen KE. The vestibular system: multimodal integration and encoding of self-motion for motor control. Trends Neurosci (2012) 35(3):185–96. 10.1016/j.tins.2011.12.001 22245372 PMC4000483

[135] Jamali M Chacron MJ Cullen KE. Self-motion evokes precise spike timing in the primate vestibular system. Nat Commun (2016) 7:13229. 10.1038/ncomms13229 27786265 PMC5095295

[136] Jamali M Mitchell DE Dale A Carriot J Sadeghi SG Cullen KE. Neuronal detection thresholds during vestibular compensation: contributions of response variability and sensory substitution. J Physiol (2014) 592(7):1565–80. 10.1113/jphysiol.2013.267534 24366259 PMC3979612

[137] Jamali M Sadeghi SG Cullen KE. Response of vestibular nerve afferents innervating utricle and saccule during passive and active translations. J Neurophysiol (2009) 101(1):141–9. 10.1152/jn.91066.2008 18971293 PMC3815216

[138] Curthoys IS Vulovic V Burgess AM Cornell ED Mezey LE MacDougall HG The basis for using bone-conducted vibration or air-conducted sound to test otolithic function. Ann N Y Acad Sci (2011) 1233:231–41. 10.1111/j.1749-6632.2011.06147.x 21950999

[139] Uchino Y Kushiro K. Differences between otolith- and semicircular canal-activated neural circuitry in the vestibular system. Neurosci Res (2011) 71(4):315–27. 10.1016/j.neures.2011.09.001 21968226

